# Low proportion of women who came knowing their HIV status at first antenatal care visit, Uganda, 2012–2016: a descriptive analysis of surveillance data

**DOI:** 10.1186/s12884-020-03197-z

**Published:** 2020-08-27

**Authors:** Miriam Nakanwagi, Lilian Bulage, Benon Kwesiga, Alex Riolexus Ario, Doreen Agasha Birungi, Ivan Lukabwe, John Bosco Matovu, Geoffrey Taasi, Linda Nabitaka, Shaban Mugerwa, Joshua Musinguzi

**Affiliations:** 1 Uganda Public Health Fellowship Program– Field Epidemiology Track, Ministry of Health – Makerere University School of Public Health, P.O. Box 7072, Kampala, Uganda; 2 AIDS Control Program, Ministry of Health, Kampala, Uganda

**Keywords:** Antenatal care, HIV testing, Known HIV status, Women, Family planning, Uganda

## Abstract

**Background:**

HIV testing is the cornerstone for HIV care and support services, including Prevention of Mother to Child Transmission of HIV (PMTCT). Knowledge of HIV status is associated with better reproductive health choices and outcomes for the infant’s HIV status. We analyzed trends in known current HIV status among pregnant women attending the first antenatal care (ANC) visit in Uganda, 2012–2016.

**Methods:**

We conducted secondary data analysis using District Health Information Software2 data on all pregnant women who came for ANC visit during 2012–2016. Women who brought documented HIV negative test result within the previous 4 weeks at the first ANC visit or an HIV positive test result and/or own HIV care card were considered as knowing their HIV status.

We calculated proportions of women with known current HIV status at first ANC visit, and described linear trends both nationally and regionally. We tested statistical significance of the trend using modified Poisson regression with generalized linear models. For known HIV positive status, we only analyzed data for years 2015–2016 because this is when this data became available.

**Results:**

There was no significant difference in the number of women that attended first ANC visits over years 2012–2016. The proportion of women that came with known HIV status increased from 4.4% in 2012 to 6.9% in 2016 and this increase was statistically significant (*p* < 0.001). Most regions had an increase in trend except the West Nile and Mid-Eastern (p < 0.001). The proportion of women that came knowing their HIV positive status at first ANC visit was slightly higher than that of women that were newly tested HIV positive at first ANC visit in 2015 and 2016.

**Conclusion:**

Although the gap in women that come at first ANC visit without knowing their HIV positive status might be reducing, a large proportion of women who were infected with HIV did not know their status before the first ANC visit indicating a major public health gap. We recommend advocacy for early ANC attendance and hence timely HIV testing and innovations to promptly identify HIV positive women of reproductive age so that timely PMTCT interventions can be made.

## Background

A known HIV status is the cornerstone for HIV prevention, treatment and support services [1, 2] including services for Prevention of Mother to Child Transmission of HIV (PMTCT) [3–5]. Prevention of mother-to-child transmission of HIV (PMTCT) is contingent on four pillars that make up the global World Health Organization (WHO) strategy for PMTCT: 1) Primary prevention of HIV among women of reproductive age which can be achieved through behavioral interventions, 2) Prevention of unintended pregnancies in women who are HIV positive, which relies on meeting the family planning needs of this population group, 3) prevention of mother to child transmission of HIV (PMTCT) through offering antiretroviral therapy (ART) to HIV-infected pregnant women and their babies and 4) care and treatment for the children that turn HIV positive through follow-up of infants born to HIV-infected mothers as well as continued care and treatment for the mothers and support to their families [6].

With an HIV prevalence among women of reproductive age of 7.6% in Uganda in 2016 [7], it is critical that HIV testing is emphasized at every possible opportunity in this sub-population group. In Uganda, HIV testing services are offered in facility and community settings, and opt-out HIV testing at first antenatal care (ANC) visit. The latter are facility-based and focus on provider-initiated testing and counseling [8].

In Uganda, where > 95% of women make at least one ANC visit, the first ANC visit has been promoted as a critical gateway for PMTCT. At first ANC visit, all women receive provider-initiated, opt-out HIV counseling and testing [5, 9, 10]. This measure has been effective in reducing mother-to-child transmission of HIV (MTCT) in Uganda [11, 12] with an 86% reduction in the number of new paediatric HIV infections during 2010–2016.

However, in Uganda women often come late for first ANC visits [13]. This coupled with increasingly lower yields of HIV positive persons at HIV testing facilities and communities with the average HIV positive yield stagnating at 3.5% among the general population in the HIV testing services program [8] undermines the national efforts to eliminate mother to child transmission of HIV. However, the low yield at ANC could be because Uganda has made strides towards achieving United Nation’s Joint Program on AIDS (UNAIDS)’ first 90, which aims at ensuring that at least 90% of all people infected with HIV know their status [14].

According to the Uganda Population HIV Impact Assessment of 2016, 94% of the HIV positive women surveyed self-reported knowing their status [15]. However, there is minimal information on the pregnant women that known their HIV status by the time they attend the first ANC visit. In this study, we sought to ascertain the proportion of pregnant women who came for the first ANC visit with known current HIV status during 2012–2016 and also compared proportions of women that came with known HIV positive status with newly identified HIV positive status at first ANC visit. This was aimed at informing the PMTCT program in Uganda of the level of achievement of UNAIDS’ first 90 by the first ANC visit. Knowledge of HIV status is associated with reduction of risky behavior and timely commencement of prevention and/or care and treatment services.

## Methods

### Study setting

Uganda is located in East Africa and is composed of 34.6 million people [16]. As of 2018, there were an estimated 9 million women of reproductive age [17]. In 2016, Uganda’s fertility rate was 5.4 children per woman [18].

Among Ugandan adults, the HIV prevalence declined from 7.3% in 2011 [19] to 6.2% in 2016 [7]. In two national surveys conducted 5 years apart, the HIV prevalence was higher among women than men (8.2% versus 6.1%) in 2011 [19] and 7.6% versus 4.7% in 2016 [7]. HIV prevalence is also higher among women living in urban areas (9.8%) than those in rural areas (6.7%) [7].

**Fig. 1:**
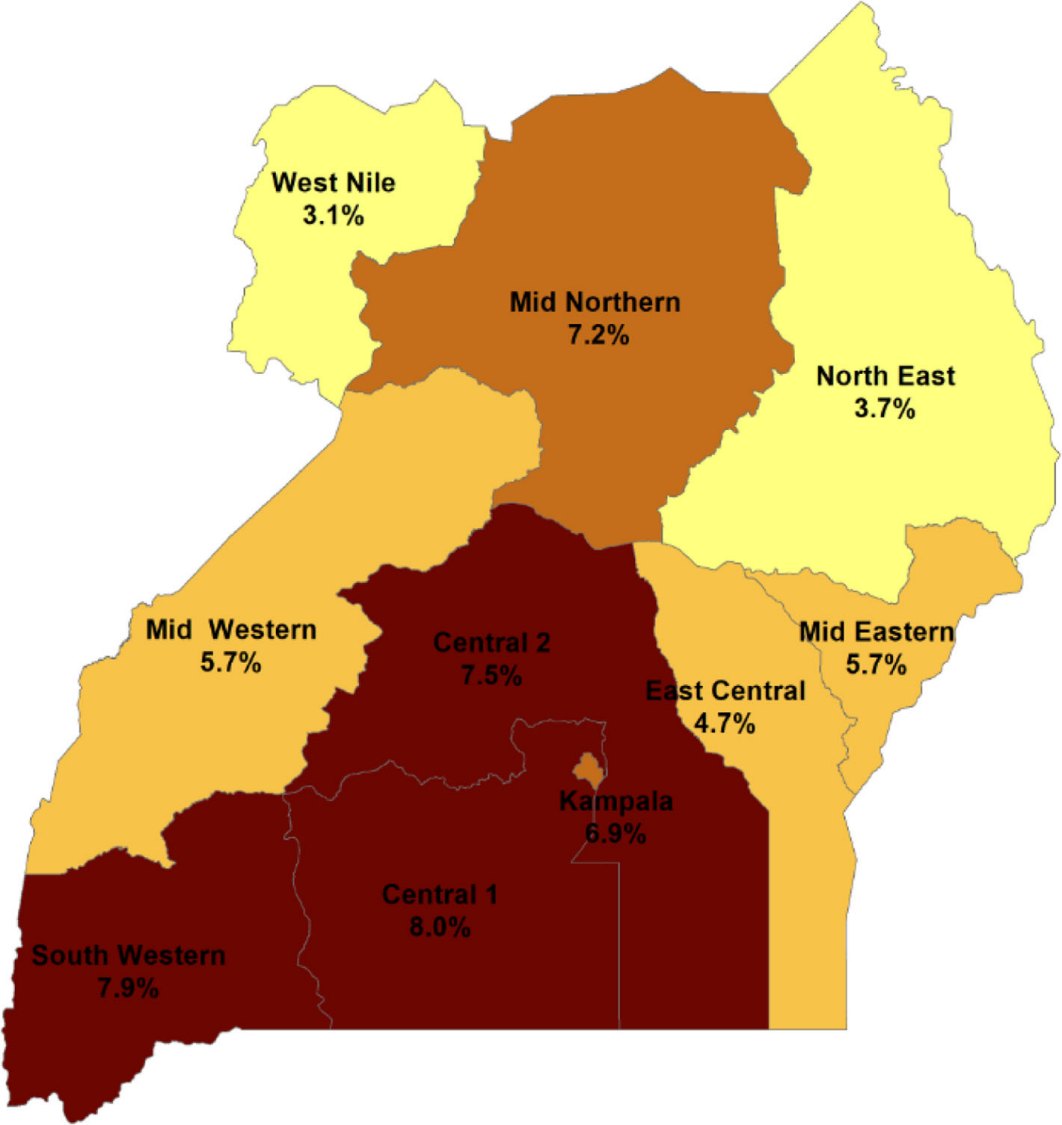
A map showing the 10 HIV regions and the respective HIV prevalence, Uganda, 2016. The map was constructed using QGIS browser 2.8.2 and the data used was from the Uganda Population HIV Impact assessment of 2016 [7]. The HIV prevalence differed in each of the 10 regions in Uganda. It was highest in Central 1 and lowest in West Nile

Administratively, Uganda was divided into 116 districts at the time of the study. These districts were categorized into 10 regions on which HIV programming was and still is based [19]. The HIV prevalence differs in each of the 10 HIV regions, with Central 1 having the highest prevalence at 8.0% and West Nile having the lowest prevalence at 3.1% [7] (Fig. 1).

### Study design

We conducted secondary data analysis of first ANC visit attendance obtained from the Uganda National Health Management Information System (HMIS). Health facilities collect information on different health variables and summarize them on various standardized HMIS forms. These are submitted to the districts, where the information is entered in the web-based District Health Information Software version 2 (DHIS2) [20]. The DHIS2 is managed by the Ministry of Health and collates data from all health facilities in Uganda.

### Data sources

Our data source was DHIS2 from the HMIS 105 report. The HMIS 105 report is an integrated monthly health facility report that contains, among other variables, monthly attendance figures for the Maternal and Child Health Services (MCH). The MCH section contains sub-sections on antenatal services including first ANC visit attendance and HIV status at first ANC visit. Starting in 2015, the indicator of ‘pregnant women who knew status before first ANC visit’ was further revised to also include ‘pregnant women who came with known HIV-positive status’ [20]. Thus the difference between ‘pregnant women who knew status before first ANC visit’ and ‘pregnant women who came with known HIV-positive status at first ANC visit’ were the pregnant women who came with known HIV-negative status at first ANC visit. Women who attended first ANC visit with documentation of an HIV negative test result obtained within the past 4 weeks were considered to have a ‘known HIV negative status at first ANC visit’ [8]. The rationale for the stringent four-week period is to be able to capture the pregnant women that might have seroconverted at the earliest opportunity. Women who had an HIV care card or a documented HIV-positive test result from a test done at any point in the past were considered to have a ‘known HIV positive status at first ANC visit’ [8].

We extracted data on total first ANC visit attendance and pregnant women who knew their HIV status at first ANC visit for all 116 districts in Uganda during 2012–2016. For known HIV positive status at first ANC visit, we could only extract data for 2015 and 2016 for the reason above. We also extracted data on pregnant women who newly tested HIV-positive for the first time at the time of their first ANC visit during 2015 and 2016.

### Data management and statistical analysis

We used Microsoft Excel, Epi info, and STATA 14 for analyses. Prior to analysis, we categorized the districts into the 10 regions used for HIV programming in Uganda, adapted from the AIDS Indicator Survey of 2010–2011 (Fig. 1) [19]. We used frequencies and proportions to report sample characteristics at national level. We calculated proportions of women with known HIV status at first ANC visit at national and regional levels and used line graphs to describe the trend of known HIV status at first ANC visit for the period 2012–2016.

We determined the annual incidence of women with known HIV status by calculating the proportion of women that came with known HIV status at first ANC visit of the total first ANC visit attendance for that year. We used the improved Poisson with generalized linear models to examine whether known HIV status at first ANC visit increased over the period under study. The known HIV status at first ANC visit was the outcome of interest and year was the independent variable. We opted for the modified Poisson regression because the outcome variable was a count - that is the number of women that came with a known HIV status at the first antenatal care visit, and we were able to obtain incident rate ratios which were a closer association between dependent and independent variables than odds ratios from logistic regression models.

We interpreted the resulting incident rate ratio (IRR) as the average change in the proportion of women who came with a known HIV status at first ANC visit and used the 95% confidence interval to ascertain significance.

We also calculated proportions of women with known HIV positive status at national and regional levels and compared these for 2015 and 2016. However, we could not describe their trends because of the very short time frame of available data of only two years.

We calculated and compared the proportions of pregnant women that tested HIV-positive for the first time at their current pregnancy at national level for 2015–2016. This was for comparability with known HIV positive status at first ANC visit in the 2 years.

## Results

### Population characteristics

The number of women that attended first ANC visit ranged from 1,431,418 in 2012 to 1,715,377 in 2016. There was no statistically significant difference in the number of women that attended first ANC visit visits over the years 2012 to 2016. HIV prevalence at first ANC visit was 2.9% in 2015 and rose to 5.9% in 2016. The proportion of women that came with known HIV status was highest at 6.9% in 2016. The proportion of women that came with a known HIV positive status at first ANC visit in 2015 and 2016 was slightly higher than that of women that were newly tested HIV positive at first ANC visit in 2015 and 2016 (Table 1).

**Table 1:** Proportion of women that came with known HIV status at first antenatal care, Uganda, 2012–2016

	2012	2013	2014	2015	2016
ANC1 attendance (N)	1,431,418	1,615,294	1,569,199	1,692,268	1,715,377
Known HIV status (n) (%)	63,270 (4.4)	96,044 (5.9)	85,208 (5.4)	104,892 (6.2)	119,082 (6.9)
HIV prevalence at ANC1 (n) (%)	ND	ND	ND	49,120 (2.9)	101,450 (5.9)
Known HIV+ status (n) (%)	ND	ND	ND	27,362 (1.6)	59,914 (3.5)
Newly tested HIV+ (n) (%)	NA	NA	NA	21,758 (1.3)	41,536 (2.4)

### National trend of known current HIV status at first ANC visit

**Fig. 2:**
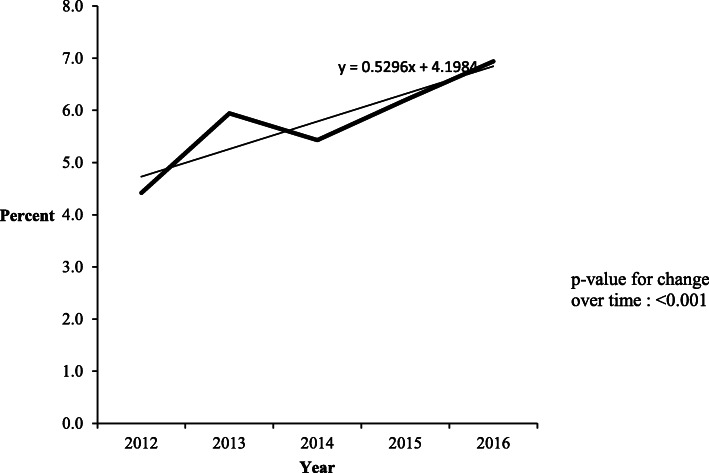
National trend of women with known HIV status at first antenatal care visit, Uganda, 2012–2016. The proportion of women who came with known HIV status at first antenatal care visit increased from 4.4% in 2012 to 6.9% in 2016

The proportion of women that came with known HIV status ranged from 63,270 (4.4% of first ANC visit attendance) in 2012 to 119,082 (6.9% of first ANC visit attendance) in 2016 (Table 1). This increase in proportion was significant (IRR = 1.14, 95% CI 1.14–1.14) (Fig. 2).

### Regional trends of known HIV status at first ANC visit

**Fig. 3:**
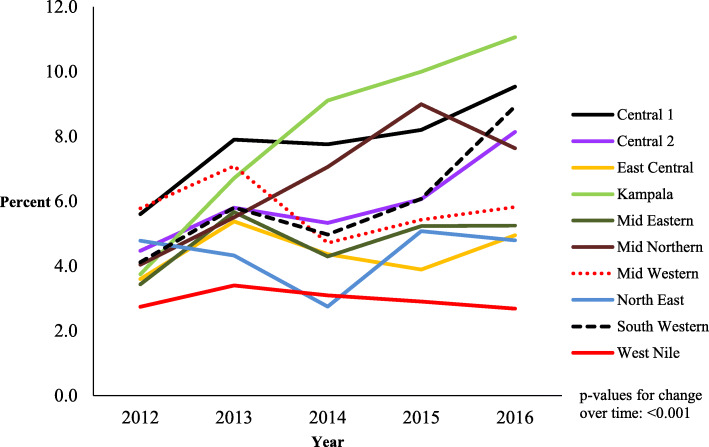
Regional trends of women with known HIV status at first antenatal care visit, Uganda, 2012–2016. Kampala registered the highest increase in proportion of women who came with known HIV status at first antenatal care visit while West Nile registered the lowest and a stagnant proportion of women that came with known HIV status at first antenatal care visit

During 2012 to 2016, the proportions of women that came with known HIV status varied in the different regions and were less than 10% in all the regions except Kampala for the years 2015 and 2016. In 2015, the proportion of women that came with a known HIV status in Kampala was 10.0% and it rose to 11.1% in 2016. Most of the regions had an increase in trend of known HIV status over the years 2012 to 2016 except the West Nile and Mid-Eastern that had a declining trend (Fig. 3). The proportion of women with known HIV status in the West Nile region stagnated at 2.7% in 2012 and 2016, while in the Mid-Eastern region ranged from 3.4% in 2012 to 5.2% in 2016.

## Discussion

Overall, although Uganda had a significant increase in the proportion of women who came to first ANC visit with known current HIV status during 2012–2016, this increase was small. There was also regional variation in trends of women coming with known HIV status at first ANC visit. The proportion of women who came to first ANC visit with known HIV status was low over the years of study, with fewer than 10% of women knowing their current status nationally. The proportion of known HIV positive status at first ANC visit is slightly higher than that of women that were newly tested HIV positive at first ANC visit in 2015 and 2016 nationally.

Although we found low proportions that came for the first antenatal visit knowing their HIV status over the years, national surveys show that more than half of Ugandans have been tested for HIV at some point in the past. The 2011 Uganda AIDS Indicator Survey showed that 83% of women and 70% of men had ever been tested and had received results of their last test [19], and information from the National HIV Testing Services showed that 42–51% of the population aged 15–49 years knew their HIV status in 2016, and that about 60% of these were women [8].

The overt difference in proportions of known HIV status found in this study compared to the national surveys were most likely due to the stringent measure of the known HIV negative status at first ANC visit which skewed the overall proportions of women that came with known HIV status at first ANC visit towards the unknown. In Uganda, one is considered to be known HIV negative at first ANC visit when the documented test was done within 4 weeks of the visit [20]. However, the stringent measure is aimed at early identification of all the HIV positive women so as to implement timely PMTCT interventions. Thus it is possible that more than 5–10% of the women attending first ANC visit knew their HIV status, but either did the test outside the required window of time or attended first ANC visit without any document verifying their status, and so were considered to be of unknown HIV status.

On a positive note, the proportion of known HIV positive status at first ANC visit was slightly higher than that of women that were newly tested HIV positive at first ANC visit in 2015 and 2016. This could be due to the nationwide progress towards achieving UNAIDS first 90 which is 90% of the HIV-positive persons in a given population knowing their HIV- positive status (14). In Uganda, between July 2015 and June 2016, 69% of persons living with HIV (PLHIV) knew their (HIV-positive) status [21] and this had increased to 73% between July 2016 and June 2017 [22]. Thus, the nationwide progress of the first 90 possibly also included the women of reproductive age.

The yield of those newly testing HIV positive at first ANC visit was less than that observed in the general population of 3.5% [8]. The yield ascertained in this study could be even further lower considering that some known HIV positive women may choose to present at first ANC visit as unknown status for a number of reasons including denial. This could be mitigated by HIV recency testing. Unfortunately, we did not have any sense of recent infections because at the time of the study, recency testing was in its pilot stages in Uganda.

The HIV positive yield obtained in this study being lower than the general population yield contradicts evidence that a big proportion of new HIV infections in Uganda are among women of reproductive age [7]. On a positive note, this could be due to the effective combination prevention efforts that have been made countrywide to reduce incident HIV cases. It could also be possible that the PMTCT program has been successful with many positive women being diagnosed through the program during previous pregnancies as well as other avenues. However, it can be due to the fact that some women do not attend ANC and thus may miss HIV testing. This calls for innovative measures to identify the ‘hidden’ new HIV positive individuals especially women of reproductive age if we are to achieve elimination of mother-to-child transmission of HIV (EMTCT).

The variations in trends in proportions of women that come with a known current HIV status at first ANC visit regionally may be attributed to the differences in the HIV prevalence in the different regions. The 2016 Uganda Population-based HIV Impact Assessment puts the highest prevalence at 7.7% in South Western region, 6.6% in Kampala and the lowest at 2.8% in West-Nile [7]. This regional variation of prevalence is similar to the one of the 2011 Uganda AIDS Indicator Survey [19]. The fact that the more highly-prevalent regions also had higher proportions of women attending first ANC visit with known current HIV status could be because HIV testing campaigns and services are more emphasized in these regions. Higher prevalence regions potentially have higher HIV positive yields [8] which in turn gives better return on investment in HIV testing. As such, more proportions of people in these regions are tested since they are perceived to be at greater risk of HIV than their counterparts in the low prevalence regions. Thus areas of low HIV prevalence such as the West Nile had more stagnating or declining trends of people that came with known HIV status at first ANC visit.

### Limitations and strengths

Our findings should be interpreted with the following limitations. We used DHIS2 data which is aggregate data and so we could not look out for individual effects such as repeat pregnancies in the same woman during the study period. Also, some variables were new and could not be assessed over the whole study period. Relatedly, the new variables (data elements) are initially not very accurate because the health workers that often double as data entrants take some time getting accustomed to looking out for and reporting them.

Our estimate of the proportion of women who knew their current HIV status at first ANC visit was likely an underestimate due to the documentation required to determine a known HIV status at first ANC visit. However, the underestimation was most likely skewed to the HIV negative women who had to have had a test within 4 weeks. The HIV positive women are less likely to be underestimated because they are more likely to report for ANC with their HIV care card.

Our analysis was only a bivariate trend. The effect of other characteristics that could have been considered in the final model such as the woman’s parity and age or whether she was a rural or urban dweller could not be assessed. This is because the data we used was aggregate and so could not account for individual characteristics. Other covariates such as government spending and donor funding would have been important to analyze, however, the donor funding by region differs from the national programming HIV regions considered in this analysis.

In addition, ANC data in DHIS2 have potential selection biases such as: distribution of public and private ANC services, misrepresentation since not all women attend ANC in DHIS2 reporting facilities and a small proportion opts not to attend professional ANC at all [23–25]. Nevertheless, a large proportion of Uganda’s population attends public health facilities [26] and so the results can be generalized to the entire country.

Finally in countries with a mature and generalized HIV epidemic such as Uganda, ANC indicators are important sources of data in HIV surveillance and provide good data on epidemic trends over time [23–25]. Our findings therefore can be used as proxy indicator of adult Ugandan women’s seeking behavior to know their HIV status, thus reflecting the national and sub-national trends of women of reproductive age who know their HIV-positive status in Uganda.

## Conclusion

Although the gap in women that come at first ANC visit without knowing their HIV status might be reducing, still a considerable proportion of women including those who were infected with HIV did not know their status before their first ANC visit HIV test, indicating a major public health gap. We therefore recommend advocacy for timely first ANC visit attendance to facilitate early HIV diagnosis.

In addition, we recommend innovative measures such as: risk assessment based door-to-door testing by lay healthcare workers to improve knowledge of HIV status pre-pregnancy and to identify pregnant women who have not enrolled in ANC, home-based couples counselling and testing, encouraging of HIV self-testing which may be particularly beneficial for sexually active adolescent girls [27] and their partners and deliberate focus on adolescent girls and young women in other HIV programs such as those for key and priority populations, assisted partner notification and orphans and vulnerable children. We also recommend resolving the barriers to HIV testing such as stigma and discrimination, test kits stock outs and healthcare worker shortages to optimize uptake of HIV testing services [27]. These need to be implemented to identify all the HIV positive women of reproductive age in order to achieve UNAIDS’ first 90 and ultimately to achieve EMTCT.

## Data Availability

The data that support the findings of this study are held at the Ministry of Health Resource Centre, Uganda but restrictions apply to the availability of these data and so are not publicly available. Data are however available from the corresponding author upon reasonable request and with permission of Ministry of Health Resource Centre, Uganda.

## Abbreviations

AIDS: Acquired Immune-Deficiency Syndrome
ANC: Antenatal care
DHIS2: District Health Information system version 2
EMTCT: Elimination of Mother to Child Transmission of HIV, first antenatal care visit, first antenatal care visit
HIV: Human Immunodeficiency Virus
HMIS: Health Management Information System
HTS: HIV Testing Services
MCH: Maternal and Child Health
MTCT: Mother-to-Child Transmission of HIV
PITC: Provider Initiated Testing and Counseling
PLHIV: People Living with HIV
PMTCT: Prevention of Mother to Child Transmission of HIV
UNAIDS: United Nations Joint program on AIDS
VCT: Voluntary Counseling and Testing
